# Factors associated with male involvement in reproductive care in Bangladesh

**DOI:** 10.1186/s12889-016-3915-y

**Published:** 2017-01-03

**Authors:** Ghose Bishwajit, Shangfeng Tang, Sanni Yaya, Seydou Ide, Hang Fu, Manli Wang, Zhifei He, Feng Da, Zhanchun Feng

**Affiliations:** 1 School of Medicine and Health Management, Tongji Medical College, Huazhong University of Science and Technology, Wuhan, 430030 Hubei China; 2 School of International Development and Global Studies, Faculty of Social Sciences, University of Ottawa, Ottawa, ON K1N 6 N5 Canada; 3 Faculty of Health Sciences, University of Ottawa, Ottawa, ON K1N 6 N5 Canada

**Keywords:** Bangladesh, Demographic and health survey, Male involvement, Maternal mortality, Reproductive health

## Abstract

**Background:**

Men’s active involvement in reproductive healthcare has shown to be positively associated with maternal and child health outcomes. Bangladesh has made appreciable progress in its pursuance of maternal mortality related goals in the framework of the MDGs. However, there remains a lot to be accomplished to realise the long-term goals for which active participation of male counterparts in reproductive care is crucial. Therefore, the objective of the present study was to investigate factors associated with male involvement in reproductive health among Bangladeshi men.

**Methods:**

We used data from Bangladesh Demographic and Health Survey (BDHS) conducted in 2011. Study participants were 1196 married men, aged between 15 and 69 years and living in both urban and rural households. Level of male involvement (outcome variable) was measured based on the responses on knowledge, awareness and practice regarding reproductive health. Chi-square tests and multivariable logistic regression models were performed for data analysis.

**Results:**

Out of 1196 participants, only 40% were found to be active about partners’ reproductive healthcare. Chi-square test showed significant association between active involvement and ever hearing about family planning (FP) in television, learning about FP through community health events, community health workers and poster/billboard. Results from logistic regression analysis revealed that type of residency [*p* = 0.004, AOR = 0.666, 95% CI = 0.504–0.879], literacy [secondary/higher education- *p* = 0.006. AOR = 0.579, 95% CI = 0.165–0.509], learning about family planning from Newspaper [*p* < 0.001. AOR = 1.952, 95% CI = 1.429–2.664], and television [*p* = 0.017. AOR = 1.514 95% CI = 1.298–1.886], and having been communicated about family planning by community health workers [*p* = 0.017. AOR = 1.946, 95% CI = 1.129–3.356] were significantly associated with active involvement of men in reproductive health issues.

**Conclusions:**

Level of male involvement was associated with schooling experience, type of residency and exposure to electronic media. National health policy programs aimed at promoting male involvement in reproductive care should focus on improving knowledge and awareness of reproductive health though community health education programs with a special focus in the rural areas.

## Background

Since the declaration of Millennium Development Goals, there has been an increased attention on women’s health in healthcare research and policymaking. As a key indicator of international development, MDG 5 was dedicated to the reduction of the maternal mortality rate by 75% by 2015. However, progress towards achievement of this goal has been inadequate, a mere 34% decline since 1990, and yet uneven across different world regions [1]. According to WHO, the developing countries, especially those in Sub Saharan Africa and Asia share a discriminate burden of maternal mortality (respectively 900 and 450 against 9 in developed regions in 2005), which remains the second largest cause of mortality among women of reproductive age in these countries. With about 85% of global population, developing countries altogether account for about 99% all maternal mortality cases [2]. Moreover, about 97% of all unsafe abortions occur in LMICs which contributes to about 15% of total maternal mortality in these countries [3]. Statistics on the utilisation of maternal health services (MHS) is equally disheartening. In the developed world about 98% women receive adequate number of ANC services, and skilled birth attendants supervising 94% of the deliveries [4]. In the LMICs in contrast, about half of all women remain deprived of adequate ANC services [5]. Two broad perspectives from which researchers attempt to explain this stark difference include the efficacy of healthcare systems such as quality, access and infrastructural barriers [6, 7], and proximate determinants such as economic, gender, health behaviour and sociocultural barriers [8, 9]. Among the themes that commonly emerge in the sociocultural context of reproductive health, violence against women (VAW) [10], and male involvement [11] have been two very important and challenging ones. In this study, we focus on male involvement and aim to explore the factors associative factors among men in Bangladesh.

The issue of male involvement in reproductive care was first pronounced officially in a conference on Population Development in Cairo held in 1994 [1]. Since then the number of empirical studies and demand for contextual evidence on sexual and reproductive health seeking behaviour and their determinants have also grown considerably. Research evidence from other South Asian countries suggests that men’s involvement in women’s reproductive care has a crucial role to play to increase the uptake of maternal health services and reduce maternal and infant mortality [12–14]. Reproductive health seeking behaviour of an individual has shown to be a psychological construct affected by various proximal/individual (perception of health, self-efficacy, motivation) [12, 14, 15] and distal/social influences (social norms and values, belief systems, degree of openness about personal matters) [16, 17]. There is also lot to accomplish especially in the areas of universal access to reproductive health services, increasing the rate of institutional delivery and adoption of family planning which have shown to be more effective in active presence of male counterparts [15, 18]. In addition to the rate of utilisation of maternal healthcare service, male participation is also positively associated with pregnancy outcomes. Prior studies have shown that male involvement was significantly associated with reduced odds of postpartum depression and improved utilisation of maternal health services [6]. In the predominantly patriarchal society as seen across the South Asian region, women in Bangladesh are generally dependent on male counterparts for making decisions on matters as general as their own and children’s healthcare, household purchases and visiting relatives [15]. Being faced with household power imbalance and having minimized control over resources would generally necessitate for even greater involvement of men in women’s health issues. Apart from that, the longstanding sociocultural view on sexual and reproductive health (SRH) is directed in a way that negatively affects reproductive health communication between partners and understanding each other’s positions regarding such matters [19, 20]. The depth of perception of reproductive health needs among men and women and their SRH seeking behavior are strongly influenced by the established meanings of reproduction embedded in the society in which they live [21]. In a qualitative study conducted on a group of Bangladeshi men, participants reported feeling uneasy to discuss reproductive health and STDs related issues with their wives, accompany them to healthcare centres and avoided dealing with reproductive health related complications with service providers [20]. Similar studies conducted in other countries have suggested in-depth population based studies to explore the underlying causes of inadequate participation of men in reproductive health. However, studies on this topic in the context of Bangladesh is remarkably scare. To this end, we conducted this research with the intention to enrich the literature and facilitate policy making aimed at promoting male involvement in maternal health in the country.

## Methods

### Data source, study area, and sampling procedure

We used the sixth round of Bangladesh Demographic and Health survey (BDHS) data for this study. The data is nationally representative, cross-sectional in nature, and carried out in 2011 from July 8 through December 27. Data were sourced from the official website of DHS (dhsprogram.com). The National Institute of Population Research and Training (NIPORT), a renowned health research organization in Bangladesh [22], conducted the survey. The survey is a part of the International Demographic and Health Survey program known as MEASURE DHS, which is currently active in about 90 countries, and conducted under the auspices of the United State Agency for International Development (USAID) and technical assistance of ICF International of Calverton based in USA.

The survey employed a two-Stage cluster sampling method covering the population residing in noninstitutional settings in Bangladesh. The two-stage clustering of the population involved labelling the smallest administrative units as enumeration areas (EAs) or clusters, each consisting of households at mouza or mohalla level. Firstly, selecting EAs based on their size proportional to that of the units. Secondly by selecting household systematically from each EA to ensure effective sampling. BDHS 2011 selected 600 EAs, however only one third were selected for men sample. In total 4,343 men were found eligible for the survey among which 3,997 were finally surveyed (response rate of 92%). More details regarding ethics protocol on biomarkers used in Demographic and Health surveys are available at: http://goo.gl/ny8T6X.

#### Subjects

Study subjects were male participants ageing between 15 and 69 years. In total 1196 men were finally included in the analysis.

#### Variables

Level of activeness of male involvement in reproductive care was the response variable in this study.

In order to select the potentially relevant covariates in the context of male involvement in reproductive health, an extensive literature review was conducted surrounding the most proximal themes: demographic and socioeconomic factors and media use status [23, 24]. Secondly, based on the availability of variables in the dataset, the following items were selected for analysis: Age, type of residency, religion, educational attainment, type of occupation, level of earning, sex of household head, number of members in the household, interaction with CHWs and in community health events, and media use (newspaper, TV and radio).

#### Measurements

Male participation was measured based on answers to a composite scoring on three items (shown in Table 1): knowledge (4 questions), awareness (3 questions), and practice (5 questions). Each correct/positive answer was assigned score ‘1’, and ‘0’ if incorrect/negative. Total score ranged from ‘0’ to’12. Based on the contrast between individual scores and population mean scores, male involvement were dichotomized as active (total score ≥ mean score of the sample), and passive (total score < mean score of the sample) [23].

**Table 1:** Percentage of participants answering correctly and being involved in maternity issues

Test questions	Frequency	Percent
Women need to have medical checkup during pregnancy	1129	94.4
During pregnancy, women need to eat more	993	83.0
Contraception is woman’s business so man should not worry	569	47.6
Month of pregnancy women need to have first check-up	270	23.0
Medical persons visited during last pregnancy	443	37.0
Wife visited health facility	557	46.6
Antenatal check-ups for the mother of most recent child	840	70.2
Husband present at birth of last child	852	71.2
Husband present during visit of medical person	435	36.4
Husband talked to wife about medical personnel	665	55.6
Husband talked with medical personnel himself	393	32.9
Husband is involved in healthcare decision making of wife	478	40

Age was trichotomised into 3 groups: 15–29 years, 30–44years, and 45–64years. Place of residency was categorized as rural and urban. Religion was categorized into Islam ‘1’ and others ‘0’ (Hinduism, Buddhism, Christianity). Educational attainment of participants were categorized into three groups based on the total number of years of receiving formal education: 0 = Nil, 1 = Primary (1–5 years), 3 = Secondary /Higher (>6 years). Type of occupation was categorized in the following way: 1) Farming = Farmer, agricultural worker, fisherman, poultry farmer, cattle raising; 2) Blue collar jobs = carpenter, mason, driver, construction worker, rickshaw puller, brick breaking, road building; 3) White collar jobs = Businessman, physician, lawyer, accountant, teacher, government service holder. Utilization of paper and electronic media has been shown to be associated with reproductive health behaviour. This study included three types of media use: TV, listening, radio, newspaper; and was dichotomized flowingly: 0 = not using at all, 1 = using occasionally/ regularly.

#### Statistical analysis

The first step in the data analysis was descriptive statistics. Percentages of study population across the independent variables were calculated. Cross tabulation was performed to identify the independent variables of significant association with the level of male participation. Significance of associations was estimated by *χ* ^2^-test. Only the variables, which showed statistical significance (*p* < 0.05) in *χ* ^2^-test were retained for regression analysis. All the explanatory variables were entered simultaneously into the regression model. Data were adjusted for sampling weight and for clustering effects. We performed intraclass correlation (ICC) analysis prior to choosing appropriate regression model. As ICC value was found insignificant, multiple regression method was performed. Finally, we conducted binary regression analysis (Generalised estimating equations) to sort out the variables which significantly impacted male participation status in reproductive care [25, 26]. Results of the regression analysis were reported in terms of *p*-values, odds ratios and 95% confidence intervals. *p*-value less than 0.05 (two tailed) was considered statistically significance in all cases. All analyses were performed using SPSS version 20.0 for MAC (SPSS Inc. Chicago. IL. USA).

## Results

Table 1 shows the frequency and percentage of correct answers by participants regarding reproductive issues. Majority of the men had correct knowledge about requirement of food and necessity of check-up during pregnancy (94.4 and 83% respectively). Only 23% of the men knew the correct timing of first check-up during pregnancy. Regarding contraception, almost half the men were of opinion that reproduction is women’s issue and does not concern men. Almost half of the men said that they did not have any idea whether or not wife visited any health facility or was visited by a medical person. However, 70.2% of them knew if wife received antenatal check-up for during pregnancy. About one-third of the participants reported being present during visit by a medical person and 71.2% present during delivery of the last child. 55.6% men discussed about medical persons with wife and about one-third communicated with medical persons himself.

### Baseline information regarding the study population

Table 2 outlines the basic characteristics of the study population (*n* = 1196). About one-third of the participants belonged to the age group of 30–44 years and more than three fifths were of rural origin. 88.9% of the sample population were Muslim which is almost the same as observed at country level (~89.5). Almost two-third of the participants completed secondary school while one-fifth received no formal education. 28.1% of the sample consisted of farming population. Proportion of both blue- and white-collar professionals were more one-third of the total sample population, however only 12.1% of the total sample reported earning sufficient income to support family. More than a quarter of the sample reported having insufficient income level. Almost all the participants were from male-headed households and more half of had 5–8 members. About half of the total participants reported having the habit of reading newspaper. Percentage of respondents watching TV and listening to radio were 92 and 16.3 respectively. Only about a quarter (27.3%) of the subjects reported ever hearing about family planning. Among the three media of information regarding family planning included in this study; poster, billboard and leaflet combined (27.3%) were the most popular compared to community health workers (7.4%) and community events (7.3%).

**Table 2:** Baseline characteristics of the study population

Variables	Frequency	Percent
Age		
15–29	383	32.0
30–44	711	59.4
45–64	102	8.5
Residency		
Rural	759	63.5
Urban	437	36.5
Educational attainment		
Nil	245	20.5
Primary	778	65.1
Secondary and above	173	14.5
Religion		
Islam	1063	88.9
Other	133	11.1
Occupation		
Farming	336	28.1
Blue collar	438	36.6
White collar	422	35.3
Earning		
Sufficient	145	12.1
Moderately Sufficient	721	60.3
Insufficient	330	27.6
Number of household members		
2–4	395	33.0
5–8	612	51.2
> 8	189	15.8
Household head		
Male	1159	96.9
Female	37	3.1
Reads news paper		
Yes	590	49.3
No	606	50.7
Watches TV		
Yes	1100	92.0
No	96	8.0
Listens to radio		
Yes	195	16.3
No	1001	83.7
Ever heard of family planning (FP) in TV		
Yes	389	32.5
No	807	67.5
Heard about FP from community health workers		
Yes	88	7.4
No	1108	92.6
Heard about FP in community events		
Yes	87	7.3
No	1109	92.7
Heard about FP from: poster/billboard/leaflet		
Yes	327	27.3
No	869	72.7

Almost all the explanatory variables were found be significantly associated (*p* < 0.05) with the level of involvement in reproductive healthcare (Table 3) and were retained for final regression analysis. Mean score was of male involvement 5.7 ± 2.2. The results show that only 40% of the participants were actively involved in women’s reproductive matters. The variables, which were excluded from regression analysis, are age, religion, number of household members and utilisation of radio.

**Table 3:** Chi-square results test showing the association between the levels of involvement in reproductive issues across the explanatory variables

Variables	Scores on level of involvement (%)		*P*
	Passive (59.9)	Active (40.1)	
Age	59.9	40.1	
15–29	32.8	30.8	0.196
30–44	57.7	62.1	
45–64	9.5	7.1	
Residency			<.001*
Rural	70.5	52.9	
Urban	29.5	47.1	
Educational attainment			<.001*
Nil	28.4	8.8	
Primary	64.0	66.7	
Secondary and above	7.7	24.6	
Religion			0.126
Islam	10.2	12.5	
Other	89.8	87.5	
Occupation			<.001 *
Farming	33.8	19.6	
Blue collar	38.5	33.8	
White collar	27.7	46.7	
Level of earning			0.001*
Sufficient	9.8	15.6	
Moderately Sufficient	59.5	61.5	
Insufficient	30.7	22.9	
Number of household members			0.493
2–4	31.8	34.8	
5–8	52.5	49.2	
> 8	15.6	16.0	
Sex of household head			0.012
Male	97.9	95.4	
Female	2.1	4.6	
Reads newspaper			<.001*
Yes	36.2	69.0	
No	63.8	31.0	
Watches TV			0.007*
Yes	89.4	95.8	
No	10.6	4.2	
Listens to radio			0.517
Yes	16.3	16.2	
No	83.7	83.8	
Ever heard of FP in TV			<.001*
Yes	25.1	43.5	
No	74.9	56.5	
Heard about FP from CHW			<.001*
Yes	4.9	11.0	
No	95.1	89.0	
Heard about FP in community events			<.001*
Yes	94.8	89.6	
No	5.2	10.4	
Heard about FP from: poster/billboard/leaflet			<.001*
Yes	21.1	36.7	
No	78.9	63.3	

*Statistically significant at *p* <0.05. *CHW* Community health worker

### Factors associated with active involvement of men in women’s reproductive health matters

Table 4 shows that male involvement was significantly associated with type of residency, level of education, reading newspaper and learning about FP from community health workers. Type of occupation, sex of household head, watching TV, listening to radio, learning about FP from community activities were not significantly associated with active involvement. Results illustrate that participants with formal education were more likely to have active participation in reproductive care compared to those with no education. Men who read newspaper were twice as likely to have active involvement. Though learning about FP from community events and poster/billboard media were found be to be associated with male involvement in Chi-square test, it showed no significant impact in regression analysis. However, odds of active involvement were also twofold among men who learned about FP form CHWs.

**Table 4:** Results of ordinal logistic regression showing factors associated with level of activeness in maternal health issues among Bangladeshi men, 2011

Variables	*B*	*p*-value	AOR (95% CI)	*p*-value	COR (95% CI)
Residency (Rural)^a^					
Urban	0.425	0.004	0.666 (0.504-0.879)	<0.001	0.654(0.633-0.676
Educational attainment (Nil)^a^					
Primary	-0,560	<0.001	0.290 (0.165-0.509)	<0.001	0.293(0.256-0.336
Secondary and higher	-1,226	0.006	0.579 (0.391-0.858)	<0.001	0.571(0.511-0.639)
Read Newspaper (No)^a^					
Yes	0.675	<0.001	1.952 (1.429- 2.664)	0.007	0.509(0.312-0.831)
Ever heard about family planning in TV (No)^a^					
Yes	0.418	0.017	1.514 (1.298-1.886)	<0.001	1.519(1.506-1.533)
Learned about FP from community health worker (No)^a^					
Yes	0.507	0.017	1.946 (1.129-3.356)	<0.001	1.661(1.270-2.172)

*AOR* Adjusted Odds Ratio, *COR* Crude Odds Ratio, *CI* Confidence Interval; (Adjusted for Occupation, Sex of household head, Level of earning, Watch Television, Learned about FP from Poster/billboard/leaflet, Learned about FP in community events)

*FP* Family planning, *CHW* Community health worker

## Discussion and policy recommendations

Results of this study showed that only 40% of the men had active involvement in reproductive care, and knowledge and awareness regarding reproductive health was remarkably low. Though most participants knew that women need institutional care during pregnancy, knowledge about timing for pregnancy checkup, contraception and awareness about utilisation of MHS by wife and rate of physical presence in service utilisation was meagre (Table 1). This result is not surprising given the result that one-fifth of the participants had no formal education and only 14.5% attained secondary or higher level education (Table 2). Previous studies have reflected on the importance of husbands’ education on positive reproductive health behaviour [11, 22, 27]. Poor knowledge concerning SRH is also shown to be associated inadequate communication about reproductive matters among family members and grow a virtual barrier for cross-gender cooperation thereby [28]. Conversely, better communication on SRH has positive impacts on reproductive health awareness [29]. Findings of our study suggest that literacy has a crucial role to play in ensuring male involvement in reproductive care which is consistent with prior studies conducted in other south Asian countries [27, 30, 31]. In Bangladesh, the reserved view towards SRH matters exist largely because there is not enough political incentive and civil society motivation to create room for the subject in the tradition health belief systems. Programs aimed at promoting male participation in reproductive must focus on systematically addressing the social barriers in a culture friendly way to ensure effectiveness and long-term success.

Type of residency also appeared to be a significant determinant of male involvement in reproductive care. The urban-rural divide regarding reproductive health behaviour is explainable by the fact that people in urban areas tend to have higher literacy and socioeconomic status, enjoy better access to healthcare service and receives greater media exposure, all of which are likely to improve health behaviour in general [32, 33]. In our study, men who reported having the habit of reading newspaper occasionally or regularly had higher participation in reproductive care. Therefore, newspaper coverage of reproductive health information is likely to generate potential benefits. However unexpectedly, we didn’t find any association between electronic media exposure such as TV and radio. This may be due to the increasing number of mobile phone subscribers, rapid expansion of internet and social networking sites, which made the traditional media less interesting especially among urban residents. Despite that, TV and radio programs remain a source of entertainment and pastime for many. In China, watching television was found to be strongly associated with adoption of modern contraceptive methods and the number of children desired [33]. As the population in Bangladesh is predominantly rural, the media sector should take innovative actions to design TV/radio entertainments more interesting and effective by incorporating health messages into age specific programs to encourage positive attitude towards reproductive health.

Another important contribution of our study is that it found a positive correlation between communication with CHWs about FP programs and male involvement in reproductive care. In Bangladesh, CHWs occupy a crucial position in the continuum of healthcare providers especially in remote areas as the country faces huge human resource deficit in healthcare and poses challenges to meet the population health needs [34]. Involvement of CHWs has proven the potential for cost-effective services in areas as critical as maternal and neonatal care [35] and DOTS for tuberculosis [36]. However, their potential remains far from being fully developed and exploited especially in the domain of reproductive care services. Apart from providing direct healthcare services, CHWs can play a vital role in implementing strategies for changing attitude towards reproductive health in both men and women. Feeing of confusion and embarrassment in physician-patient communication is a common thing while discussing confidential matters among young patients. CHWs can bridge the gap substantially since they are usually recruited from the same environment. As they already have some degree of understanding and intimacy with the local populace, people have the advantage of expressing themselves more easily and thus creating the climate for positive attitude and behaviour towards reproductive health [29].

Results also indicate that men who learned about FP from CHWs are more likely to be involved in reproductive care which is consistent with the prior studies showing the association between SRH education and positive attitude towards reproductive health behaviour [20, 37]. Bangladesh government has made several programmatic efforts to enhance community-based educational intervention programs to promote maternal and infant health. However, such programs to enhance reproductive health knowledge would require a different approach to ensure participation of both men and women. Educational programs targeting women’s health education were found to be effective in improving their knowledge and reproductive health behaviour [38, 39]. Studies have found that SRH educational programs had greater impact on maternal health behaviors when both spouses are involved compared to when only women participated the program [40]. This finding is supported by the fact that SRH behaviour is actually shaped more effectively by social and institutional interactions instead of individual learning [14] warrants for increased focus on improving learning by interaction and sharing of information through community based health events. Community programs bear special significance for Bangladesh since school-based reproductive health education program is not yet developed. The consequence runs at household level as parents with inadequate knowledge regarding reproductive health also show reservations towards communicating reproductive issues with children [28] which presents major constraints towards improving reproductive health knowledge and communication among peers. Community based programs has to be tailored in a way to tackle such obstacles that are not yet implemented in schools e.g. creating positive attitude among parents.

Besides its contribution to the current literature, this study has few mentionworthy limitations. Firstly, we used secondary data, which meant that we had no control in selecting the variables and the way they were measured. Secondly, male involvement was measured in terms of performance on knowledge, awareness and practice levels which are subjective matters and prone to misreporting by the participant and hence may not represent the actual scenario. The DHS survey was conducted in 2011, and prevalence of several factors (literacy rate, level of knowledge and awareness, media use status) might have changed since then.

## Conclusions

The factors that can influence the degree male participation in reproductive care can vary according to the sociocultural environment in which individuals live and interact. Based on a nationally representative data DHS in Bangladesh, our study concludes that educational (years of schooling, access to electronic media e.g. TV & radio) and community level factors (communicating with community health worker about FP) play important roles in male involvement in the country. Given an understaffed and underfunded healthcare system, it is suggested that policy makers pay special attention to organizing health education campaigns through engaging CHWs targeting men especially in rural areas to improve knowledge and attitudes towards reproductive care.

## Abbreviations

CHWs: Community health worker
DSH: Demographic and health survey
FP: Family planning
MHS: Maternal health services
MMR: Maternal mortality rate
SBA: Skilled birth attendants
SRH: Sexual and reproductive health
